# Erratum to “Is the lockdown important to prevent the COVID-19 pandemic? Effects on psychology, environment and economy-perspective” [Ann. Med. Surg. 56 (2020) 38–42]

**DOI:** 10.1016/j.amsu.2020.07.001

**Published:** 2020-07-09

**Authors:** Abdulkadir Atalan

**Affiliations:** Department of Industrial Engineering, Gaziantep Islam, Science and Technology University, 27010, Gaziantep, Turkey

The publisher regrets that this paper was processed as a Short Communication ‘Perspective’, when it should have been Original Research. This has now been amended and published as Original Research.

The publisher would like to apologise for any inconvenience caused.

